# Supplementary material for: ‘Navigating a lonely road to adulthood with an ever-changing map’; a framework and case study protocol exploring the transition to adulthood, mental health literacy and occupational balance among Swedish young adults
Source: PLoS One. 2025 Jul 22;20(7):e0327617. doi: 10.1371/journal.pone.0327617 (PMC12282861; doi:10.1371/journal.pone.0327617)
Supplement: S2 Appendix — (DOCX) [file pone.0327617.s002.docx]

**Interview Guide – Focus Group Interviews with Young Adults**‘Navigating a lonely road to adulthood with an ever-changing map’; A Framework and Case Study Protocol Exploring the Transition to Adulthood, Mental Health Literacy and Occupational Balance among Swedish Young Adults

Martin Karaba Bäckström, Sonya Girdler, Ulf Jonsson, Carita Håkansson, Annika Lexén

**Main Research Question:**

What does the transition to adulthood mean for young adults in Sweden, and how does it affect their mental health, activity balance, and support needs? How do they perceive their own mental health literacy?

Occupational balance means having a balance between different activities in daily life, such as work, leisure, sleep, and home/household responsibilities. It also includes having a balance between activities of different types, such as voluntary/mandatory and energy-giving/energy-draining activities.

Mental health literacy is defined as knowledge and understanding of mental health problems that influence the recognition, management, and prevention of such problems. It includes recognizing signs of mental illness, knowledge of available treatments, self-help strategies, and understanding how to support others showing signs of mental ill-health.

| Main Questions | *(Possible follow-up questions)* |
| --- | --- |
| Create a word cloud via mentimeter.com. Ask participants to write three words about what the transition to adulthood means to them. | *Discuss and ask follow-up questions based on the words that appear in the cloud.* |
| - *How does this affect your mental health?* - *Can you share your experience of transitioning to adulthood?* | - Have you noticed any specific challenges among friends/peers during the transition to adulthood? If so, which ones? - How have these challenges affected their well-being? |
| - What physical, emotional, and social changes have you experienced? | - How have these changes affected your mental well-being? Please give examples. |
| - When you think about everything you do in your daily life, how do you perceive the balance between your various activities?  Create a Menti poll | - - How do you experience the balance between different activities in daily life such as work/studies, leisure, sleep, home/household?   - How would you estimate the number of hours you spend in each category per week?   - How do you perceive the balance between activities you have to do and those you want to do?   - How do you perceive the balance between activities that drain and give you energy?   - Is there anything that makes it difficult for you to maintain the balance you want?   - How many hours per day do you estimate you spend on doom-scrolling? How does that feel? |
| - How would you describe your own knowledge of mental health/ill-health? - *PROMOTING ONE'S OWN MENTAL HEALTH* - *Recognizing signs of mental illness* - *Knowledge of available treatments and self-help strategies* - *Understanding how to support others who show signs of mental illness* | - Is there any knowledge you feel you are missing? - Do you know how to support a friend who isn’t feeling well or shows signs of mental illness? If yes, how? Please give examples. |
| - What support do you think is important for young adults to manage the challenges that may arise during the transition to adulthood? |  |
| - What do you think is important for promoting mental health among young adults? Please give examples. |  |
