# Supplementary material for: ‘Navigating a lonely road to adulthood with an ever-changing map’; a framework and case study protocol exploring the transition to adulthood, mental health literacy and occupational balance among Swedish young adults
Source: PLoS One. 2025 Jul 22;20(7):e0327617. doi: 10.1371/journal.pone.0327617 (PMC12282861; doi:10.1371/journal.pone.0327617)
Supplement: Peer review history

# Editor report: Decision Letter 0

**DOI:** 10.1371/journal.pone.0327617.r001

**Authors:** Avanti Dey

‘Navigating a lonely road to adulthood with an ever-changing map’; A Framework and Case Study Protocol Exploring the Transition to Adulthood, Mental Health Literacy and Occupational Balance among Swedish Young Adults

PONE-D-25-31103

Dear Dr. Karaba Bäckström,

We’re pleased to inform you that your manuscript has been judged scientifically suitable for publication and will be formally accepted for publication once it meets all outstanding technical requirements.

Within one week, you’ll receive an e-mail detailing the required amendments. When these have been addressed, you’ll receive a formal acceptance letter and your manuscript will be scheduled for publication.

An invoice will be generated when your article is formally accepted. Please note, if your institution has a publishing partnership with PLOS and your article meets the relevant criteria, all or part of your publication costs will be covered. Please make sure your user information is up-to-date by logging into Editorial Manager at Editorial Manager®  and clicking the ‘Update My Information' link at the top of the page. If you have any questions relating to publication charges, please contact our Author Billing department directly at authorbilling@plos.org.

If your institution or institutions have a press office, please notify them about your upcoming paper to help maximize its impact. If they’ll be preparing press materials, please inform our press team as soon as possible -- no later than 48 hours after receiving the formal acceptance. Your manuscript will remain under strict press embargo until 2 pm Eastern Time on the date of publication. For more information, please contact onepress@plos.org.

Kind regards,

Avanti Dey, PhD

Staff Editor

PLOS ONE

Additional Editor Comments (optional):

We have deemed your submission eligible for expedited acceptance based on the documents provided, and the quality of your work. Prior to submitting the final version of your manuscript, please clarify whether your prospective search terms as outlined in lns 508-511 are comprehensive - if not, please include a full comprehensive list of search terms that will be used in your study, either directly in the text or as a Supplemental Table/Figure.

Thank you for submitting your work to PLOS ONE!

Reviewers' comments:

# Editor report: Acceptance letter

**DOI:** 10.1371/journal.pone.0327617.r002

**Authors:** Avanti Dey

PONE-D-25-31103

PLOS ONE

Dear Dr. Karaba Bäckström,

I'm pleased to inform you that your manuscript has been deemed suitable for publication in PLOS ONE. Congratulations! Your manuscript is now being handed over to our production team.

At this stage, our production department will prepare your paper for publication. This includes ensuring the following:

* All references, tables, and figures are properly cited

* All relevant supporting information is included in the manuscript submission,

* There are no issues that prevent the paper from being properly typeset

You will receive further instructions from the production team, including instructions on how to review your proof when it is ready. Please keep in mind that we are working through a large volume of accepted articles, so please give us a few days to review your paper and let you know the next and final steps.

Lastly, if your institution or institutions have a press office, please let them know about your upcoming paper now to help maximize its impact. If they'll be preparing press materials, please inform our press team within the next 48 hours. Your manuscript will remain under strict press embargo until 2 pm Eastern Time on the date of publication. For more information, please contact onepress@plos.org.

If we can help with anything else, please email us at customercare@plos.org.

Thank you for submitting your work to PLOS ONE and supporting open access.

Kind regards,

PLOS ONE Editorial Office Staff

on behalf of

Dr. Avanti Dey

Staff Editor

PLOS ONE
